# The relationship between smartphone addiction and anxiety: a cross-sectional study among Moroccan nursing students

**DOI:** 10.11604/pamj.2025.50.47.45274

**Published:** 2025-02-11

**Authors:** Rachida Archou, Meriem Ouadrhiri, Mounia Amazian, Nawal Mouhoute, Driss Touil, Rachid Aalouane, Kamelia Amazian

**Affiliations:** 1 Clinical Neuroscience Laboratory, Faculty of Medicine and Pharmacy, Sidi Mohammed Ben Abdellah University, Fez, Morocco,; 2 Laboratory of Health Sciences, Care and Techniques, Higher Institute of Nursing Professions and Health Techniques, Fez, Morocco,; 3 Biomedical and Translational Research Laboratory, Faculty of Medicine and Pharmacy, Sidi Mohammed Ben Abdellah University, Fez, Morocco,; 4 Higher Institute of Nursing Professions and Health Techniques, Rabat, Morocco,; 5 Research Laboratory in Management Sciences, Mohammed V University of Rabat, Rabat, Morocco,; 6 Communication Department, CHU Hassan II, Fez, Morocco,; 7 Higher Institute of Nursing Professions and Health Techniques, Kenitra, Morocco,; 8 Psychiatry Department, Ibn Al Hassan Hospital, CHU Hassan II, Fez, Morocco,; 9 Human Pathology, Biomedicine and Environment Laboratory, Faculty of Medicine and Pharmacy, Sidi Mohammed Ben Abdellah University, Fez, Morocco

**Keywords:** Smartphone addiction, anxiety, relationship, nursing students, Morocco

## Abstract

**Introduction:**

nursing students face serious problems related to smartphone addiction. Anxiety is among the problems associated with this addiction. In Morocco, this relationship still needs to be investigated. The objective of this study was to examine the relationship between smartphone addiction and anxiety and analyze the factors related among Moroccan nursing students.

**Methods:**

this is a cross-sectional study. The data was collected using a self-administered questionnaire that included items on socio-demographic characteristics, smartphone use, and lifestyle behaviors. The smartphone addiction scale short version and the Beck Anxiety Inventory were used.

**Results:**

three hundred and thirty-eight (308) students took part in the study. The prevalence of smartphone addiction was 41.2%, 42.4% for females, and 39.5% for males without significant association. For anxiety, 34.1% have severe anxiety and 21.1% have moderate anxiety. In the multivariate analysis, anxiety was found to be a factor associated with smartphone addiction (OR=1.33; 95% CI: 1.04, 1.70; p=0.02). Two other independent factors were also found to be associated with smartphone addiction, which is residence with parents (OR=1.68; 95% CI: 1.01, 2.78; p=0.05) and smartphone consultation in the morning (OR=0.80; 95% CI: 0.61, 0.96; p=0.02). The participants with high levels of anxiety have the highest smartphone addiction scores with a statistically significant association (p=0.000).

**Conclusion:**

this study showed significant levels of smartphone addiction and anxiety among the participants. A relationship between these two variables has been highlighted. It is essential to educate these young students in terms of the rational use of smartphones to avoid any negative influence on their daily lives.

## Introduction

Recently, smartphones have become widely used in virtually every sphere of society [1]. They are everywhere and, for some, an indispensable ally [2]. However, despite its many advantages, the increasing use of smartphones can lead to over-consumption or addiction, especially among young people [1,3-5]. Indeed, smartphone addiction has become a real public health problem that particularly affects young people [6].

Effectively, in China, a study carried out among medical students revealed a prevalence of smartphone addiction of 48.16% [7]. In Malaysia, among medical students, the prevalence of smartphone addiction was 40.6% [8]. In a study among university students in Saudi Arabia, the prevalence of smartphone addiction among participants was 67.0% [9]. In Tunisia, a study at the medical school level showed that 55% of participants had a smartphone addiction [10]. In Morocco, a study carried out in 2017 among a sample of young adults revealed a prevalence of smartphone addiction of 55.8% [11]. These results show that more than a quarter of higher education students presented an addiction to smartphones, which implies that the study of the factors and consequences on the health of students is necessary [8].

In this sense, several authors explain that such addiction is likely to lead to an increase in mental health problems among young people [12,13]. Moreover, anxiety is one of the mental problems most associated with smartphone addiction [14]. Indeed, there is evidence to suggest that smartphone addiction may be closely related to anxiety [15,16].

For nursing students, the demands of clinical training make them a specific population whose smartphone use may differ from that of other disciplines [17]. Additionally, promoting the improvement and stability of nursing students' mental health and well-being is essential [18]. Furthermore, high smartphone addiction scores were observed among nursing students with negative effects on various dimensions of life [19]. Indeed, the prevalence of smartphone addiction is high among nursing students with the existence of a close relationship between this addiction and anxiety symptoms [20]. Likewise, psychologically, smartphone addiction among nursing students is associated with an increase in anxiety [3,17]. Moreover, a survey conducted in Brazil among nursing students revealed that there was a correlation between symptoms of depression, anxiety stress, and smartphone addiction. However, this relationship is complex and its essence is not yet defined [20].

In this context, the purpose of this study was to examine the relationship between smartphone use and anxiety symptoms in a sample of Moroccan nursing students because the likelihood of smartphone addiction is increasing among young students. Very few studies were performed on nursing students and that leads to the selection of this topic [3,17,19,20].

## Methods

**Study design:** this is a cross-sectional study that adopts an approach aimed at determining the relationship between smartphone addiction and anxiety among Moroccan nursing students. The study was conducted in April 2023.

**Setting:** the study site was the Higher Institute of Nursing and Health Techniques (ISPITS) of Fez, Meknes annex. It is a higher education institution that does not belong to universities and is under the supervision of the government authority responsible for Health. It participates in the effort to integrate, coordinate, and rationalize the national system of higher education. This Institute ensures the preparation and delivery of national diplomas organized in three cycles of study in the fields of Nursing Professions and Health Techniques (Professional License Cycle, Master's Cycle, and Doctoral Cycle). The ISPITS are organized into seven headquarters institutes to which 16 annexes are attached [21].

**Participants:** the survey targeted all bachelor's degree students for the 2022-2023 academic year any option and any level combined and who number 446 students. The participants were recruited using the convenience sampling method where the subjects were chosen because of their accessibility and the proximity of the researchers.

**Inclusion criteria:** i) all students present at the time of the survey; ii) all students who voluntarily agreed to participate in the study.

**Exclusion criteria:** i) students with serious health problems; ii) students who did not use smartphones.

**Study size:** a convenience sampling was adopted for the study taking into consideration the accessibility criterion. Using the formula:


$$N=\frac{\frac{z^{2}\times p\left(1-p\right)}{e^{2}}}{1+\left(\frac{z^{2}\times p\left(1-p\right)}{e^{2}N}\right)}$$

Where, N= population size; z= z-score; e= margin of error; p= standard deviation. The minimum sample size to keep was 274.

**Data sources/measurement:** data collection was carried out using a pre-tested self-administered questionnaire. The students were invited to voluntarily participate in the survey during a first contact established at the level of the higher institute of nursing professions and health techniques of Fez, annex Meknes. Thus, a first meeting with the students was established during the inter-course breaks to share with them information about the objective of the study, its progress, the data collection instrument, and the ethical considerations adopted throughout the process of carrying out the study. The students who gave their consent to participate in the study were then invited to a 2^nd^ meeting to receive the printed questionnaire and complete it. The questionnaire was administered by 4 members of the research team. Thus, all participants were assessed using the Smartphone Addiction Scale (SAS- SV) and the Beck Anxiety Inventory (BAI). The questionnaire also included sections on participants' socio-demographic characteristics, as well as smartphone use.

The SAS-SV is designed to measure the level of risk of smartphone addiction and to distinguish high-risk groups. A 6-point Likert scale is used to assess 10 items, with 1 being strongly disagree and 6 being strongly agree. As a general rule, total scores fluctuated between 10 and 60, with a higher score suggesting problematic smartphone use. The prevalence of addiction was assessed taking gender differences into account: 31 and 33 (out of 60) were selected to be classified as “male and female excessive smartphone users” respectively [22]. For the BAI, it consists of 21 self-reported items that assess the intensity of physical and cognitive anxiety symptoms over the previous week, using a four-point scale. Anxiety levels can range from 0 to 63, with: 0 to 7 = low anxiety, 8 to 15 = mild anxiety, 16 to 25 = moderate anxiety, and 26 to 63 = severe anxiety [23].

**Statistical methods:** descriptive analysis was carried out for all study variables. Categorical variables are presented as frequency (%), while continuous variables are presented as mean ± standard deviation (SD). Groups were compared using Pearson's chi-square test, Student's test, and ANOVA test. Multivariate logistic regression models were used to identify independent associated with smartphone addiction. Variables included in the multivariate analysis are those that were found to be significant in the univariate analysis at a level of p≤0.2. The statistical significance was set at p≤0.05. Statistical analyses were performed using SPSS statistical software (version 26.0).

**Ethical consideration:** participants were fully informed of the study's objectives, and gave their consent for their participation in the study and the use of the data collected. The protection of personal data was ensured throughout the study by rigorous measures designed to preserve the anonymity and confidentiality of the information provided by respondents. Prior authorization was requested from the management of the Meknes Institute before data collection began. This research and its study protocol were validated and approved by the ethics committee of the Hassan II University Hospital of Fez, under the N° 18/22.

## Results

**Participants:** of all the students at the institute, 308 agreed to take part in the study with a response rate of 69.1%.

**Descriptive data:** the mean and standard deviation of the participants' ages were 21.14 ± 2.65 years. Of all study participants, 184 (59.7%) were women and 124 (40.30%) men. 58.8% of students have lived with their parents during their studies and 68.8% have a medium socio-economic level. In terms of academic results, 42.9% of the participants had passed with honors, 38.6% did sport and 6.8% suffered from health problems. Of the participants, 8.4% smoked, 3.2% drank alcohol, 2.3% used drugs, 53.6% used gambling and 12.3% use video games excessively. During school days, 22% of the participants use their smartphone for more than 4 hours and during vacations, 48% use it for more than 4 hours (Table 1).

**Table 1:** general characteristics of participants (n=308)

	[Mean ± SD* (n)] or n (%)		[Mean ± SD* (n)] or n (%)
**Age (year)**	21.14 ± 2.65 (308)	**Smoking**	
		Yes	26 (8.4)
		No	282 (91.6)
**Sex**		**Drug use**	
Female	184 (59.7)	Yes	7 (2.3)
Male	124 (40.30)	No	301 (97.7)
**Economic level**		**Alcohol consumption**	
Low	64 (20.8)	Yes	10 (3.2)
Medium	212 (68.8)	No	298 (96.8)
High	32 (10.4)		
**Residence with parents**		**Games of chance**	
Yes	181 (58.8)	Yes	165(53.6)
No	125 (40.6)	No	143 (46.4)
**Mention**		**Video games**	
Standard pass	26 (8.4)	Often	38 (12.3)
Honours	119 (38.6)	Exceptionally	270 (87.6)
High honours	132 (42.9)		
Highest honours	21 (6.8)		
**Presence of disease**		**Duration of use during school days**	
Yes	21 (6.8)	≤ 4 hours	244 (79)
No	287 (93.2)	> 4 hours	64 (22)
		**Duration of use on vacation days**	
		≤ 4 hours	160 (52)
		> 4 hours	148 (48)

* Standard deviation

**Figure 1:**
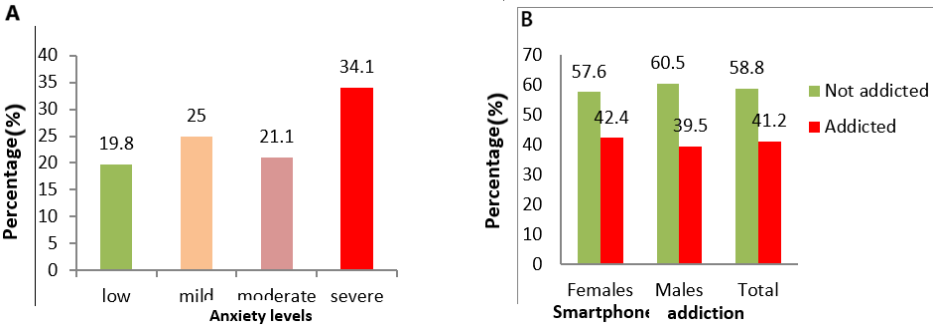
A) the Beck anxiety inventory, and B) prevalence of the smartphone addiction scale-short version

**Prevalence of smartphone addiction and anxiety among participants:** the prevalence of smartphone addiction among study participants was 41.2%. 42.4% for females and 39.5% for males without significant association. For the prevalence of anxiety among study participants, 34.1% had severe anxiety while 21.1% had moderate anxiety (Figure 1).

**Smartphone addiction and its relationship with anxiety and other associated factors:** referring to the results of univariate analysis, it was observed that residence with parents (OR= 1.74; 95% CI: 1.10, 2.77), playing sports (OR= 1.59; 95% CI: 1.002, 2.55), presence of disease (OR= 3.19; 95% CI: 1.05, 9.71), smoking (OR= 3.20; 95% CI: 1.74, 8.74), drug use (OR= 4.32; 95% CI: 0.51, 36.33), smartphone consultation in the morning (OR= 0.79; 95% CI: 0.64, 0.98), put down the phone in the evening (OR= 1.22; 95% CI: 0.89, 1.67) and anxiety (OR= 1.51; 95% CI: 1.23, 1.88) were significantly associated with smartphone addiction (Table 2). Based on the final model, the independently significant factors associated with smartphone addiction are residence with parents (OR=1.68; 95% CI: 1.01, 2.78; p=0.05), smartphone consultation in the morning (OR=0.80; 95% CI: 0.61, 0.96; p=0.02) and anxiety (OR=1.33; 95% CI: 1.04, 1.70; p=0.02) (Table 3). Participants with low or mild levels of anxiety had a minimum smartphone addiction score equal to 10 while those with moderate and severe levels of anxiety had a maximum smartphone addiction score equal to 15. Knowing that the smartphone addiction score was found to be significantly associated with anxiety levels with p=0.000 (Table 4).

**Table 2:** factors associated with smartphone addiction: results of univariate analysis

	Not addicted n (%)	Addicted n (%)	OR* (95% CI**)	p-value
**Sex**			0.88 (0.56- 1.41)	0.61
Female	106 (57.6)	78 (42.4)		
Male	75 (60.5)	49 (39.5)		
**Mention**			0.78 (0.57- 106)	0.30
Standard pass	13 (50)	13 (50)		
Honours	67 (56.3)	23.7 (55)		
High honours	77 (58.3)	55 (41.7)		
Highest honours	16 (76.2)	5 (23.8)		
**Economic level**			0.84 (0.56- 1.28)	0.50
Low	37 (57.8)	27 (42.2)		
Medium	122 (57.5)	90 (42.5)		
High	22 (68.8)	10 (31.3)		
**Alcohol consumption**			1.67 (0.42-6.56)	0.50
Yes	7 (70)	3 (30)		
No	174 (58.4)	124 (41.6)		
**Games of chance**			1.31 (0.83-2.07)	0.24
Yes	102 (61.8)	63 (38.2)		
No	79 (55.2)	64 (44.8)		
**Video games**			0.67 (0.34-1.32)	0.24
Often	19 (50)	19 (50)		
Exceptionally	102 (60)	108 (40)		
**Duration of use during school days**			1.002 (0.57-1.76)	0.90
≤ 4 hours	144 (58.8)	101 (41.2)		
> 4 hours	37 (58.7)	26 (41.3)		
**Duration of use on vacation days**			1.05 (0.67-1.66)	0.82
≤ 4 hours	95 (59.4)	65 (40.6)		
> 4 hours	86 (58.1)	62 (41.9)		
**Put down the phone in the evening**			1.22 (0.89-1.67)	0.21
I fall asleep using it	41 (59.4)	28 (40.6)		
Less than an hour before going to bed	91 (63.6)	52 (36.4)		
More than an hour before bed	49 (51.0)	47 (49.0)		

* Odds ratio, ** confidence interval

**Table 3:** factors associated with smartphone addiction: results of univariate analysis and multivariate logistic regression analysis

	Not addicted n (%)	Addicted n (%)	OR* (95% CI)	P-value	OR* (95% CI**)	P-value
**Residence with parents**			1.74 (1.10- 2.77)	**0.02**	1.68 (1.01- 2.78)	**0.05**
Yes	117 (64.6)	64 (35.4) 61 (48.8)				
No	64 (51.2)					
**Playing sports**			1.59 (1.002- 2.55)	**0.05**		
Yes	78 (65.5)	41 (34.5)				
No	103 (54.5)	86 (45.5)				
**Presence of disease**			3.19 (1.05- 9.71)	**0.03**		
Yes	17 (81)	4 (19)				
No	164 (57.1)	123 (42.9)				
**Smoking**			3.20 (1.74- 8.74)	**0.02**		
Yes	21 (80.8)	5 (19.2)				
No	160 (56.7)	122 (43.3)				
**Drug use**			4.32 (0.51-36.33)	**0.14**		
Yes	6 (85.7)	1 (14.3)				
No	175 (58.1)	126 (41.9)				
**Smartphone consultation in the morning**			0.79 (0.64-0.98)	**0.03**	0.80 (0.61- 0.96)	**0.02**
As soon as I open my eyes	41 (48.2)	44 (51.8)				
Within 15 minutes of waking up	62 (63.3)	36 (36.7)				
15 to 30 min after waking up	30 (53.6)	26 (46.4)				
More than 30 minutes after waking up	48 (69.6)	21 (30.4)				
**Anxiety**			1.51 (1.23-1.88)	**0.00**	1.33 (1.04- 1.70)	**0.02**
Low	45 (73.8)	16 (26.2)				
Mild	55 (71.4)	22 (28.6)				
Moderate	30 (46.2)	35 (53.8)				
Severe	51 (48.6)	54 (51.4)				

* Odds ratio, ** confidence interval

**Table 4:** smartphone addiction score and anxiety levels

		Smartphone addiction score		
	n	Median (min-max)	X̄*±SD**	p-value
**Anxiety**				0.000
Low	61	25(10-50)	25.54±8.84	
Mild	77	24(10-49)	26.14±10.30	
Moderate	65	33(15-50)	32.20±8.78	
Severe	105	31(14-50)	31.58±9.26	

X̄*Sample mean, **standard deviation

## Discussion

In our sample, the prevalence of smartphone addiction was 41.2%. Like the results of our study, two other studies carried out in Morocco in 2018 and 2021 among medical students revealed that smartphone addiction was 50.94% and 37.9%, respectively [24,25]. Also, in a study among students in health science in Nepal, smartphone addiction was around 36.8% [5]. Another study in India among dental students found that 42.60 % are addicted to smartphones [26]. Similarly, in Saudi Arabia, a prevalence of smartphone addiction of 48% was observed among university students [27]. Moreover, among students of medical faculties in Serbia, the prevalence of smartphone addiction was only 21.7% [28]. These observed variations may be related to differences between the participants and the environment in which they live.

In the present study, there is no significant difference between the two sexes which is consistent with the results of a study among undergraduate university students in Liban where smartphone addiction is not linked to gender [29]. While a cross-cultural study of Chinese and German students showed that there were significant gender differences [30]. Also, according to a survey of health science students in Nepal, more men than women were found to be smartphone addicts [5].

For the duration of smartphone use by students, 22% of respondents use their smartphone for more than 4 hours in school days and 48% use it for more than 4 hours in vacations without significant association with smartphone addiction. These results contradict those demonstrated by a study among dental students in India where the duration of smartphone use was one of the important indicators of smartphone dependency [26]. In addition, in a study in Serbia, a significant association was observed between spending more than 4 h daily on smartphones and smartphone addiction [28]. Similarly, among undergraduate students at Medical College in Nepal, Smartphone addiction was linked to use for more than 5 hours a day on weekdays [5].

Also, the results of the present study showed that smartphone addiction is significantly associated with residence with parents. Indeed, students living far from their parents were more at risk of smartphone addiction. Similarly, a study carried out in Turkey among university students showed that students living away from their families have high levels of smartphone addiction [31]. However, in a study carried out in Serbia, there was no association between smartphone addictions and living with parents during studies [28].

In our study, we observed a statistically significant association between smartphone addiction and smartphone consultation in the morning. However, the nature of this relationship remains ambiguous. In this context, a study among Lebanese students showed that using a smartphone is the first thing on their minds when they wake up in the morning [29]. The prevalence of anxiety observed in our study aligns with findings from previous research, which consistently reports high anxiety levels among nursing students [32-34]. Additionally, we identified a significant association between smartphone addiction and anxiety, corroborating results from several other studies. Thus, among health sciences students, higher levels of anxiety are associated with smartphone addiction [35]. In addition, a study conducted with Iranian university students has highlighted the association between anxiety and smartphone addiction [36]. Another study among medical students in China also showed that smartphone addiction was associated with various psychological and behavioral problems, such as anxiety [37]. Similarly, in a study among Indian dental students, anxiety was one of the factors that hurt the mental well-being of users addicted to smartphones [26]. In addition, smartphone addiction was positively associated with anxiety in a study conducted among university students in China [38].

Additionally, the highest smartphone addiction scores were observed in participants with moderate or severe anxiety levels, which is consistent with the results of a study conducted with university students in Nigeria where smartphone-addicted users scored higher in terms of anxiety symptoms [39]. Likewise, in a study carried out in Malaysia, the students who reported high scores of smartphone addiction tended to report high scores of anxiety [6]. Similarly, a study in Turkey showed that there were significantly positive correlations between anxiety levels and SAS scores [40].

In sum, there is currently little data available to provide conclusive evidence for a comprehensible categorization of smartphone addiction. However, the comparison of this addiction with the classification of excessive behaviors can be equivalent in terms of severity to already established addictions such as problematic computer use or excessive gaming [41].

**Limitations:** the present study has certain limitations which may influence the results obtained. The cross-sectional nature of the study only indicates an association. In addition, the participants in the study came from the Higher Institute of Nursing Professions and Health Techniques in Meknes, which may compromise the representativeness of the sample and the external validity of the study. Furthermore, the results of this study are based on self-reported data, which implies a risk of recall bias or social desirability bias.

## Conclusion

Smartphone addiction has become a widespread issue, particularly among young people. Through the present study, it was observed that this addiction also concerns nursing students. Anxiety is one of the disorders linked to smartphone overuse, and our findings emphasize this relationship. It is important to make young people aware of these risks and to help them adopt healthy and rational use of this technology. This may include defining clear rules for smartphone use, promoting appropriate socio-cultural activities, using specialized therapies to adopt healthy behaviors or managing possible consequences. Overall, further research is needed to examine the various social, mental, and physical challenges associated with smartphone use.

### What is known about this topic

- Smartphone addiction has become a real public health problem;
- Smartphone addiction is likely to lead to an increase in mental health problems among young people;
- Existence of a close relationship between this addiction and anxiety symptoms.

### What this study adds

- Smartphone addiction is present among Moroccan nursing students;
- This study found a significant association between smartphone addiction and anxiety among Moroccan nursing students;
- This study showed there were significantly positive correlations between the SAS scores and anxiety levels among Moroccan nursing students.
